# Early-life blockade of NMDA receptors induces epigenetic abnormalities in the adult medial prefrontal cortex: possible involvement in memory impairment in trace fear conditioning

**DOI:** 10.1007/s00213-019-05362-5

**Published:** 2019-10-25

**Authors:** Joachim Latusz, Marzena Maćkowiak

**Affiliations:** Maj Institute of Pharmacology, Polish Academy of Sciences, Department of Pharmacology, Laboratory of Pharmacology and Brain Biostructure, Smętna Str. 12, 31-343 Kraków, Poland

**Keywords:** Schizophrenia, Histone, Arc, Fear memory, HDAC5

## Abstract

**Rationale:**

Several findings indicate that early-life dysfunction of N-methyl-d-aspartate (NMDA) receptors might cause schizophrenia-like abnormalities in adulthood that might be induced by impairments in epigenetic regulation.

**Objectives:**

In the present study, we investigated whether postnatal blockade of NMDA receptors (within the first 3 weeks of life) by the competitive antagonist CGP 37849 (CGP) might affect some epigenetic markers in the adult medial prefrontal cortex (mPFC).

**Methods:**

Histone H3 phosphorylation at serine 10 (H3S10ph), histone H3 acetylation at lysine 9 or 14 (H3K9ac or H3K14ac, respectively), or expression of histone deacetylase (HDAC) 2, HDAC5, myocyte enhancer factor (MEF) 2D and activity-regulated cytoskeleton-associated protein (Arc) were analysed. Moreover, we also evaluated whether the deacetylase inhibitor sodium butyrate (SB; 1.2 mg/kg, ip) could prevent behavioural and neurochemical changes in the mPFC induced by CGP during memory retrieval in the trace fear conditioning paradigm.

**Results:**

The results showed that CGP administration increased the number of H3S10ph nuclei but did not affect H3K9ac and H3K14ac or HDAC2 protein levels. However, CGP administration altered the HDAC5 mRNA and protein levels and increased the mRNA and protein levels of MEF2D. CGP also increased Arc mRNA, which was correlated with an increase in the amount of Arc DNA bound to MEF2D. SB given 2 h after training prevented impairment of the freezing response and disruption of epigenetic markers (H3S10ph, HDAC5, MEF2D) and Arc expression during memory retrieval induced by CGP administration.

**Conclusions:**

The early-life blockade of NMDA receptors impairs some epigenetic regulatory processes in the mPFC that are involved in fear memory formation.

## Introduction

N-methyl-d-aspartate (NMDA) receptors are known to be involved in synaptic maturation and circuit formation during the prenatal and early postnatal stages of brain development, and this period usually corresponds with NMDA receptor subunit switching (Snyder and Gao 2013). Changes in the NMDA receptor composition make the brain vulnerable to genetic and environmental risk factors (Snyder and Gao 2013), and the impairment of NMDA receptor function by the pharmacological blockade of NMDA receptors during a critical developmental window in rodents (the first 3 weeks of postnatal life) disturbs normal brain development and causes subsequent long-lasting behavioural, anatomical and neurochemical abnormalities in adulthood that are similar to those observed in patients with schizophrenia (Hardingham and Do 2016, Snyder and Gao 2013). For example, NMDA receptor hypofunction in animal models induced deficits in learning and memory, attention or sensory gating that mimic the cognitive symptoms of schizophrenia. Moreover, the reduction in the volume of some brain regions, i.e., the prefrontal cortex, the hippocampus, the decrease in dendritic spine density or changes in parvalbumin expression reported in patients in schizophrenia, is also observed in a neurodevelopmental model of schizophrenia based on NMDA receptor dysfunction (du Bois and Huang 2007, Hardingham and Do 2016, Snyder and Gao 2013). An important role of NMDA receptors in schizophrenia onset is that NMDA receptor hypofunction in adolescence or adulthood also leads to neurochemical and behavioural malfunctions common in schizophrenia (Hardingham and Do 2016, Snyder and Gao 2013). Thus, NMDA receptor dysfunction at any stage of development induces the emergence of schizophrenia, and the NMDA receptor hypofunction hypothesis of schizophrenia has been formed (Snyder and Gao 2013).

There is still an open question regarding the mechanisms subserving the maintenance of impairments in NMDA receptor function induced by early-life events. Recent findings indicate a role for epigenetic regulation in sustaining long-lasting changes by affecting gene transcription (Millan 2013). Epigenetic control of gene expression involves several mechanisms related to the chemical modification of DNA and histone proteins (i.e., histone acetylation, phosphorylation, methylation) or the regulatory actions of small nuclear RNAs and microRNAs (Millan 2013). Several findings indicate that impairment of post-translational histone modification in schizophrenia could be an important epigenetic mechanism involved in the pathology of schizophrenia. For instance, hypoacetylation of histone H3 at lysine 9 (H3K9ac) and lysine 14 (H3K14ac) in the prefrontal cortex in young subjects with schizophrenia has been observed (Tang et al. 2011). In addition, the expression of histone deacetylase (HDAC) 2, which regulates histone acetylation, was decreased in the dorsolateral prefrontal cortex of patients with schizophrenia in post-mortem and PET neuroimaging studies (Gilbert et al. 2019, Schroeder et al. 2017). Moreover, an increased level of phosphorylation of histone H3 at serine 10 (H3S10ph) in nucleated peripheral blood cells of schizophrenic subjects was found (Sharma et al. 2015). However, the involvement of the NMDA receptor in epigenetic dysfunction related to schizophrenia is still under investigation. The results from an animal model of schizophrenia suggest that blockade of the NMDA receptor at different stages of development, i.e., in adolescence (Aoyama et al. 2014, Koseki et al. 2012) or adulthood (Mackowiak et al. 2013), induces several changes in epigenetic regulators, such as histone H3 acetylation and phosphorylation or HDAC5 level in the adult medial prefrontal cortex (mPFC). However, whether early-life NMDA receptor dysfunction causes epigenetic impairments in adulthood has not been determined.

Therefore, in the present study, we used a neurodevelopmental model of schizophrenia based on postnatal administration (first 3 weeks of postnatal life) of the competitive NMDA receptor antagonist CGP 37849 (CGP) (Latusz et al. 2017) and investigated the effects of early-life disruption of NMDA receptors in the critical developmental window for NMDA receptor maturation (Snyder and Gao 2013) on epigenetic regulation in the adult rat mPFC. The regime of postnatal administration of a competitive NMDA receptor antagonist used in our previous studies yielded schizophrenia-like abnormalities in adulthood, i.e., sensorimotor gating deficits, impairments in working memory, reduction of social contacts, fear memory deficits (Latusz et al. 2017, Wedzony et al. 2008b) and anatomical changes in the mPFC (Wedzony et al. 2005b). We used a neurodevelopmental model of schizophrenia because of its better aetiology and emergence of schizophrenia compared with animal models based on pharmacological interventions in adulthood (Tsuang 2000). Moreover, this model showed permanent schizophrenia-like abnormalities (Wedzony et al. 2008) compared with acute or even repeated use of NMDA antagonists (ketamine, MK-801, phencyclidine) in adulthood (Adell et al. 2012, Jentsch and Roth 1999) that better reproduce changes in schizophrenia patients. In addition, an active isomer of the NMDA receptor antagonist used in the present study did not induce schizophrenia-like behaviour in adult animals (Zajaczkowski et al. 2003). Thus, the pharmacological profile of CGP and its early-life administration might also determine whether early postnatal dysfunction of the NMDA receptor induced by NMDA antagonist without psychotic properties of non-competitive NMDA antagonist (developmental models based on administration ketamine, MK-801, phencyclidine) might induce alterations in some epigenetic regulators in the adult mPFC. Thus, we determined histone modifications activating gene transcription, H3K9ac, H3K14ac and H3K10ph, as previous findings indicate their impairments in the adult mPFC in animal models of schizophrenia based on NMDA receptor dysfunction in adolescence (Aoyama et al. 2014, Koseki et al. 2012) or in adulthood (Mackowiak et al. 2013). In addition, we also analysed HDACs that regulate histone acetylation, namely, HDAC2 and HDAC5, which are related to NMDA receptor function. HDAC2 is a member of histone deacetylase class I (Millard et al. 2017) that is enriched in promoters of NMDA receptor subunits and involved in the regulation of memory formation (Guan et al. 2009). HDAC5 is a member of histone deacetylase class IIa (Parra 2015), and its expression and function are dependent on NMDA receptor activation (Chawla et al. 2003, McKinsey et al. 2001). Moreover, changes in HDAC5 levels were observed in the adult mPFC of animals with adolescent NMDA receptor hypofunction (Aoyama et al. 2014, Koseki et al. 2012) and have been shown to play a role in responses to chronic stimuli (Renthal et al. 2007), the regulation of long-term potentiation (Guan et al. 2002) and memory formation (Agis-Balboa et al. 2013). HDAC5 is recruited to its target genes through interactions with transcription factors, i.e., myocyte enhancer factor 2 (MEF2), to repress target gene transcription (Parra and Verdin 2010). MEF2 family members (MEF2A, MEF2B, MEF2C, MEF2D) regulate the expression of several genes, including activity-regulated cytoskeleton-associated protein (Arc) (Dietrich 2013, Flavell et al. 2008). Thus, the expression of MEF2D and its related gene Arc have also been studied in the adult mPFC of postnatal CGP-treated rats.

Cognitive dysfunctions are a core of schizophrenia deficits and are related to prefrontal cortex abnormalities (Lesh et al. 2011, Lesh et al. 2013, Sakurai et al. 2015). Several findings indicate that the prefrontal cortex is engaged in memory formation in trace fear conditioning (TFC) (Han et al. 2003, Runyan et al. 2004), and a deficit in fear memory in this model might suggest an impairment in prefrontal cortex function and in associative learning and memory (Raybuck and Lattal 2014). Moreover, memory deficits in TFC have been found to be present in animal models of schizophrenia based on pharmacological blockade of NMDA receptors in either early life (Latusz et al. 2017) or adulthood (Bolton et al. 2012). Although several findings indicate that fear memory function is related to epigenetic control (Fischer et al. 2007, Jarome and Lubin 2014), the involvement of epigenetic mechanisms in the prefrontal cortex in control memory formation in TFC is still under investigation (Sui et al. 2012). Thus, it was of interest to investigate whether early-life NMDA receptor dysfunction might affect epigenetic mechanisms, i.e., histone H3 phosphorylation and acetylation or HDAC levels in the adult mPFC during the memory test in the TFC. Data show that the non-specific HDAC inhibitor sodium butyrate (SB) mainly targets all class I HDACs and most class II HDACs (including HDAC5) (New et al. 2012), and acting in the mPFC regulates memory retention in TFC (Sui et al. 2012). Thus, in this study, we also determined whether systemic administration of SB is capable of inhibiting memory deficits in TFC induced by early-life blockade of the NMDA receptor. Moreover, the effect of SB administration on epigenetic dysregulation in the adult mPFC related to fear memory impairment induced by early-life NMDA receptor malfunction was also investigated.

## Materials and methods

### Animals and treatment

Pregnant dams (Wistar Han rats) were obtained from an animal provider (Charles River, Germany) at 15 days of pregnancy and housed individually in polycarbonate cages (26.5 × 18 × 42 cm). Rat pups were injected subcutaneously (sc) with vehicle (0.9% NaCl) or increasing doses of CGP 37849 [(E)-(±)-2-amino-4-methyl-5-phosphono-3-pentenoic acid] (Tocris, UK): 1.25 mg/kg on days 1, 3, 6 and 9, followed by 2.5 mg/kg on days 12, 15 and 18 and a final dose of 5 mg/kg administered on day 21 as described previously (Latusz et al. 2017). The offspring were weaned 22 days after birth. Only males were used in our experiments, since the CGP model was established only on males (Latusz et al. 2017, Wedzony et al. 2005a, b, Wedzony et al. 2008a, b). The rats were housed in groups of 4 with ad libitum access to food and water and an artificial 12/12-h light/dark cycle (lights on at 7 a.m.). The biochemical studies (Western blot analysis, quantitative real-time polymerase chain reaction (qRT-PCR), chromatin immunoprecipitation (ChIP) and immunohistochemistry) were performed on the mPFC of adult animals at postnatal days (P) 65–70 that were treated early with VEH or CGP. In adulthood, the animals were divided into two different experimental groups: (1) maintained in the home cages without any experiments (untrained animals) or (2) subjected to memory retrieval in TFC (trained animals). The experimental protocols were approved by the Committee for Laboratory Animal Welfare and Ethics of the Institute of Pharmacology at the Polish Academy of Sciences in Kraków, and the protocols met the requirements of the European Council Guide for the Care and Use of Laboratory Animals (86/609/EEC). All efforts were made to minimise animal suffering and to reduce the number of animals used.

### RNA extraction and qRT-PCR

The mPFC was dissected from coronal sections, frozen with liquid nitrogen and stored at − 20 °C. RNA extraction and qRT-PCR were performed as described previously (Mackowiak et al. 2014). qRT-PCR was performed using a QuantStudio 12 K Flex Real-Time PCR System (Applied Biosystems, USA). The gene-specific primers and probes for Arc (assay Rn00571208_g1), Hdac5 (assay Rn01464245_m1) and Mef2d (assay Rn00578329_m1) from the TaqMan Gene Expression Assays (Applied Biosystems, USA) were used, and 1 μl of each sample was used for cDNA amplification. The amplification, which used TaqMan Universal PCR Master Mix (Applied Biosystems), was conducted under the following conditions: 50 °C for 2 min and 95 °C for 10 min followed by 40 cycles of 95 °C for 15 s and 60 °C for 1 min. The expression of the glyceraldehyde-3-phosphate dehydrogenase (Gapdh) transcript (assay Rn99999916_s1), which showed stable mRNA levels, was quantified to control for variations in cDNA levels. All qRT-PCR experiments were performed in duplicate on two different occasions and included no-template controls. The cycle threshold values were calculated automatically. Relative quantification was performed using the comparative threshold (CT) method according to 2^−ΔCT^, where ΔCT = (CT, target gene − CT, reference gene).

### Chromatin immunoprecipitation

Chromatin was isolated from the mPFC using a standard protocol (EZ-Magna ChIP Merck, Germany). The extracted chromatin was sheared to 100–1000 bp using a Sonic Dismembrator Vibra-Cell VCX-130 (Sonics, USA). Each sample was sonicated five times on ice for 10 s each time at 20% of maximum power (Bator et al. 2018). Chromatin was immunoprecipitated using the antibody anti-myocyte enhancer factor 2D (MEF2D, Santa Cruz Biotechnology, USA) and EZ-Magna ChIP according to the manufacturer’s instructions. qRT-PCR was performed with primers specific to the Arc promoter. The forward primer sequence was 5′ AGAACCTTGCAGGAGCCTTA 3′, and the reverse primer sequence was 5′ ATGGAGGAACCTCAACATGG 3′ (Choi et al. 2015). Input and immunoprecipitated DNA amplification reactions were run in duplicate in the presence of SYBR Green (iQ SYBR Green Supermix, Bio-Rad, Poland). All primer sets had comparable amplification efficiencies. CT values were normalised to input DNA and to a negative control region that was not enriched for histone modifications. Data were calculated as percentages of the control.

### Western blotting

The mPFC was dissected from coronal sections, frozen with liquid nitrogen and stored at − 20 °C (Bator et al. 2018, Mackowiak et al. 2014). Cytosolic and nuclear extracts of the isolated brain region were obtained using a ProteoExtract Subcellular Proteome Extraction Kit as recommended by the manufacturer (Calbiochem, Germany). The immunoreactivity of the protein HDAC5 and its phosphorylated form (pHDAC5) were analysed in the cytosolic and nuclear fractions, while MEF2D, H3K9ac, and H3K14ac proteins were analysed in the nuclear fractions from the same animals. Protein extracts (5 μg protein/lane) were separated using 7.5% or 12% SDS-PAGE and transferred to nitrocellulose membranes using an electrophoretic transfer system (Bio-Rad). The blots were incubated overnight at 4 °C with the following primary antibodies: rabbit anti-histone deacetylase 5 (HDAC5, 1:1000, Cell Signaling Technology, USA), rabbit anti-phospho-HDAC5 (pSer259) (pHDAC5, 1:1000, Sigma-Aldrich), mouse anti-histone deacetylase 2 (HDAC2, 1:1000, Cell Signaling Technology), goat anti-myocyte enhancer factor 2D (MEF2D, 1:500, Santa Cruz Biotechnology), rabbit anti-acetyl histone H3 Lys9 (H3K9ac, 1:1000, Cell Signaling Technology), rabbit anti-acetyl histone H3 Lys14 (H3K14ac, 1:1000, Millipore), rabbit anti-histone H3 (1:25,000, Upstate/Millipore) and rabbit anti-glyceraldehyde-3-phosphate dehydrogenase (GAPDH, 1:5000, Cell Signaling Technology). Immune complexes were identified using the appropriate peroxidase-conjugated secondary antibodies against rabbit IgG or mouse IgG (1:1000, Roche, Poland) and goat IgG (1:50000, Sigma-Aldrich, Poland). The reactions were visualised with enhanced chemiluminescence (ECL; Lumi-LightPlus Western Blotting Kit, Roche), which was recorded using a luminescent image analyser (Fujifilm LAS-1000, Fujifilm Corporation Japan). The relative levels of immunoreactivity were quantified using ImagePro Plus (Media Cybernetics, USA) software. The levels of all markers were normalised to GAPDH protein, and H3K9ac and H3K14ac protein levels were also controlled by the total histone H3 protein.

### Immunohistochemical studies

Tissue preparation was performed as described previously (Mackowiak et al. 2013). Free-floating sections from the mPFC were processed for H3S10ph staining. The brain sections were incubated for 1 h in blocking buffer (5% normal goat serum (Vector Laboratories, USA) and 0.3% Triton X-100 in 0.01 M PBS) and subsequently incubated (48 h at 4 °C) with primary rabbit anti-phospho-histone H3 (Ser10) (1:750, Millipore) diluted in 3% normal goat serum and 0.3% Triton X-100 in 0.01 M PBS. The reactions were visualised with biotinylated anti-rabbit (Vector Labs) and avidin-biotin-horseradish peroxidase complex (Vectastain Elite ABC Kit, Vector Labs) at the concentration recommended by the manufacturer with a 3,3′-diaminobenzidine tetrahydrochloride. Images were captured on an Aperio Scan Scope system (UK) at objective 20, and the final photomicrographs were composed using Adobe Photoshop (Adobe Systems, USA). The number of H3S10ph-immunopositive cells in the mPFC was estimated using unbiased stereological methods as described previously (Mackowiak et al. 2013).

### Trace fear conditioning

The experimental procedure was similar to that described previously (Latusz et al. 2017) and was performed in the TSE Fear Conditioning System (Germany). On day 1, the rats were placed individually in a conditioning box for 20 min and then returned to their home cages. On day 2, the animals were returned to the conditioning boxes and underwent a 10-min baseline period followed by six trials of trace fear conditioning, where a foot shock (1 s at 0.5 mA) was delivered 18 s after the tone ended (85 dB, 2 kHz, 16 s). The intervals between tones were 198 s. During conditioning, each chamber was cleaned with a 1% solution of acetic acid. After the last trial, the rats were removed from the conditioning chamber and returned to their home cages, where they either were left undisturbed or were injected with 0.9% NaCl or SB (1.2 mg/kg, ip, Sigma-Aldrich) immediately or 2 h after training. On day 3, the rats experienced the tone (cue) test. The animals were placed in testing boxes (45 × 45 × 40 cm) made of black acrylic (permeable by infrared light) with a grey plastic floor (environmentally distinct from the conditioning box). The test arena was cleaned with 70% ethanol and was faintly illuminated (3 lux). The test was conducted with the room light off, whereas the training was conducted with the room light on. The test procedure was as follows: after a 3-min baseline period, the rats underwent three testing trials, with each trial consisting of a tone (85 dB, 2 kHz, 30 s) followed by a 60-s interval. After the cue test, the rats were returned to their home cages. The duration of freezing behaviour (in which animals remained motionless, except for respiratory movements) was automatically measured using Fear Conditioning Software (TSE, Germany).

For biochemical experiments, the rats were prepared as described above, but NaCl or SB was injected only 2 h after training when the animals were in their home cages. The rats were killed immediately after the memory retrieval test.

### Statistics

The results are presented as the group mean ± the standard error of the mean (SEM). Statistical evaluations were performed using Statistica software and consisted of one-, two- or three**-**way analyses of variance and one- or two-way repeated measures analyses of variance (ANOVAs), as indicated in the figures, followed by the Newman-Keuls post hoc test. Differences were considered to be significant at *p* < 0.05.

## Results

### Effect of early-life CGP treatment on epigenetic factors in the mPFC of adult (P65–P70) untrained animals

#### Histone modifications

To determine whether early-life blockade of the NMDA receptor might affect histone modification related to NMDA receptor function in adulthood, we analysed histone H3 phosphorylation and acetylation.

##### Phosphorylation of histone H3

We used H3S10ph immunostaining and stereological analysis to study histone H3 phosphorylation and the distribution of H3S10ph-positive cells in the whole mPFC and the cortical subdivisions: cingulate (CG), prelimbic (PrL) and infralimbic (IL).

**Fig. 1:**
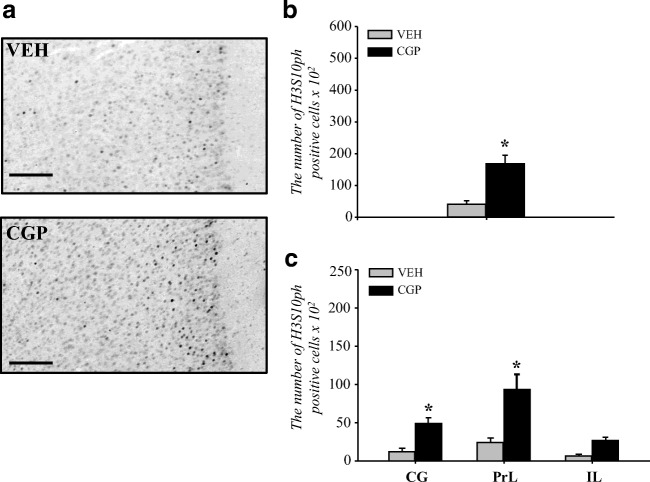
Histone H3 phosphorylation at serine 10 (H3S10ph) in the adult mPFC of postnatal CGP-treated untrained rats. Light photomicrographs of the prelimbic part of the mPFC immunoprobed for H3S10ph (**a**) and examined for the number of immunopositive cells (**b**, **c**) are shown. The number of H3S10ph nuclei in the whole mPFC (**b**) and in certain regions (cingulate (CG), prelimbic (PrL) and infralimbic (IL) cortices (**c**)). The scale bars represent 100 μm. Each data point represents the mean ± SEM; *n* = 7. *p < 0.05 vs VEH (one-way ANOVA (**b**) or one-way repeated measures ANOVA followed by the Newman-Keuls test (**c**))

The constitutive presence of H3S10ph immunostaining in the nuclei of some cells in the mPFC was noticed (Fig. 1a). CGP administration increased the number of cells positive for the presence of H3S10ph protein by almost 4-fold in the whole mPFC (*F*(1, 12) = 22.98, *p* < 0.0005; Fig. 1b).

A comparable number of H3S10ph nuclei was present in all regions of the mPFC of the VEH group (cingulate, prelimbic and infralimbic cortices, Fig. 1c). However, CGP treatment affected the number of H3S10ph nuclei dependent on subdivision of the mPFC (*F*(2, 24 = 5.67, *p* < 0.01), and a statistically significant increase in the number of H3S10ph nuclei was observed in the cingulate (CG, *p* < 0.03) and prelimbic (PrL, *p* < 0.0002) but not infralimbic (IL, *p* = 0.34) cortices (Fig. 1c).

##### Acetylation of histone H3

**Fig. 2:**
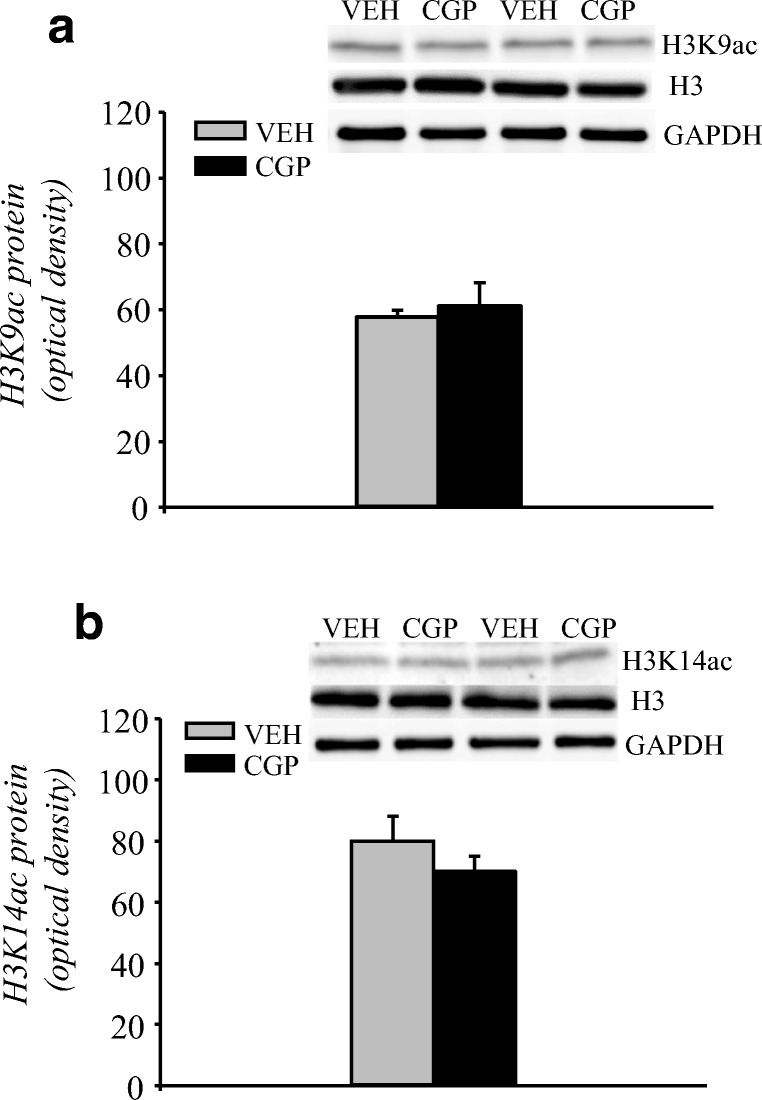
Histone H3 acetylation in the adult mPFC of postnatal CGP-treated untrained rats. Histone H3 acetylation at lysine 9 (H3K9ac; **a**) or at lysine 14 (H3K14ac; **b**) in the nuclear fraction. Photomicrographs show examples of the immunoblots of H3K9ac, H3K14ac, total histone H3 and GADPH antibodies. Each data point represents the mean ± SEM; *n* = 6 per group

Two forms of histone acetylation were investigated, H3K9ac and H3K14ac, and their protein levels were measured in the nuclear fraction of the mPFC. CGP administration did not affect the acetylation of H3K9 protein (*F*(1, 10) = 0.25, *p* = 0.63; Fig. 2a). A lack of CGP effect was also observed in the case of H3K14ac protein (*F*(1, 10) = 3.25, *p* = 0.10; Fig. 2b).

#### Histone deacetylase

We also investigated whether early-life blockade of the NMDA receptor might affect HDAC protein levels. Because of the potential role of HDAC2 and HDAC5 in the schizophrenia development (Aoyama et al. 2014, Gilbert et al. 2019, Koseki et al. 2012, Schroeder et al. 2017), we analysed their protein levels in untrained CGP-treated adult rats. The chosen HDACs are different in their catalytic activity and distribution in cell compartments (New et al. 2012).

##### HDAC2 protein

**Fig. 3:**
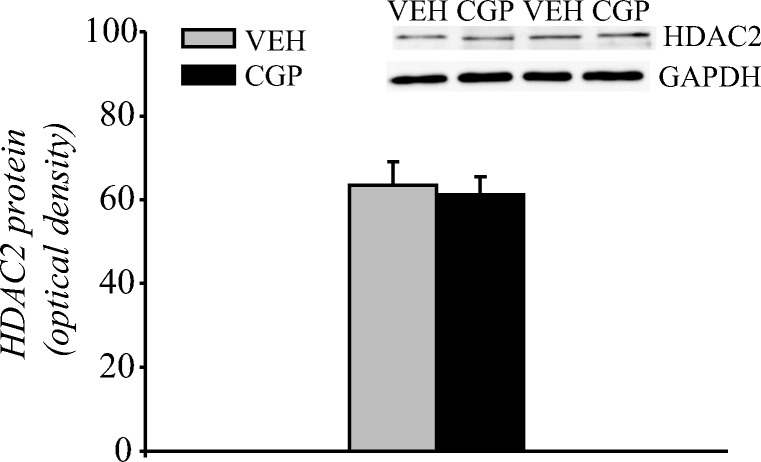
Histone deacetylase 2 (HDAC2) protein levels in the nuclear fraction of the adult mPFC of postnatal CGP-treated untrained rats. Each data point represents the mean ± SEM; *n* = 6 per group

HDAC2 protein is localised in the nuclear compartment of the cells. Thus, HDAC2 levels were analysed only in the nuclear fraction of the adult mPFC. The results showed that early-life CGP administration did not affect HDAC2 levels (*F*(1, 10) = 0.11, *p* = 0.75; Fig. 3).

##### HDAC5 protein and mRNA levels

**Fig. 4:**
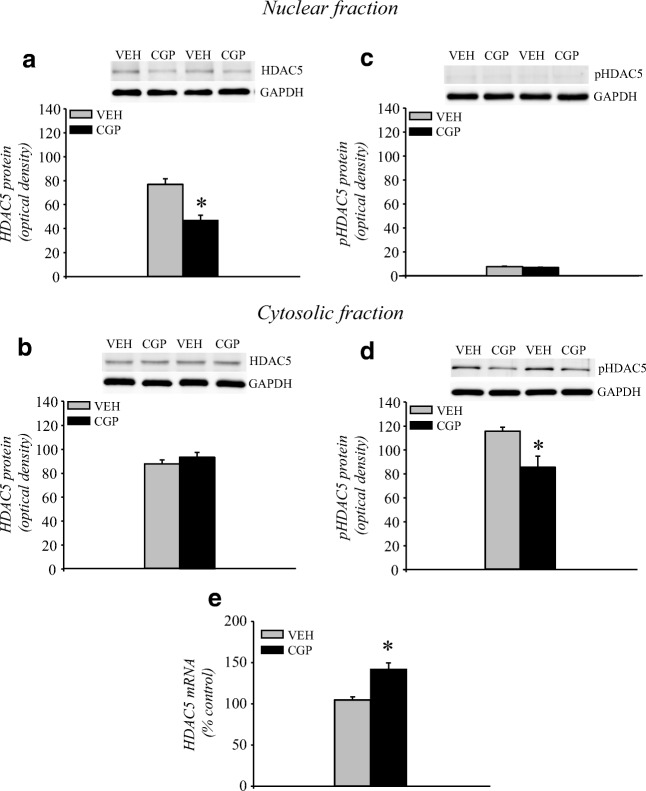
Histone deacetylase 5 (HDAC5) protein and mRNA levels in the adult mPFC of postnatal CGP-treated untrained rats. HDAC5 protein levels in nuclear (a) and cytosolic (b) fractions. Phosphorylated form of HDAC5 (pHDAC5) protein levels in nuclear (c) and cytosolic (d) fractions. Photomicrographs show examples of the immunoblots of HDAC5, pHDAC5 and GADPH antibodies. HDAC5 mRNA level (e). Each data point represents the mean ± SEM; *n* = 6 per group. *p < 0.05 vs VEH (one-way ANOVA)

HDAC5 protein is localised in both nuclear and cytoplasmic compartments, and it can shuttle between compartments in its phosphorylated form (pHDAC5). Thus, the levels of HDAC5 and pHDAC5 proteins were analysed in nuclear and cytosolic fractions of the adult mPFC (Fig. 4a, b, c, d). Postnatal CGP administration induced a decrease in HDAC5 protein levels in the nuclear fraction (*F*(1, 10) = 38.47, *p* < 0.001; Fig. 4a) but not in the cytosolic fraction (*F*(1, 10) = 1.367, *p* = 0.27; Fig. 4b). Extremely low levels of pHDAC5 immunoreactivity (at the limit of detection) were observed in the nuclear fractions of both VEH- and CGP-treated animals, and the postnatal blockade of NMDA receptors did not alter the levels of pHDAC5 (*F*(1, 10) = 4.08, *p* = 0.07; Fig. 4c). However, CGP administration decreased the levels of pHDAC5 protein in the cytosolic fraction (*F*(1, 10) = 44.24; *p* < 0.007; Fig. 4d). Because of the changes in HDAC5 protein levels, the mRNA level of HDAC5 was also analysed. CGP administration increased HDAC5 mRNA levels (*F*(1, 10) = 23.41, *p* < 0.0007; Fig. 4e).

##### HDAC5-related genes

HDAC5 expression was affected in adult mPFC by early-life blockade of NMDA receptor; thus, it was also interesting to determine whether the HDAC5-related transcription factor MEF2D and linked to MEF2D Arc expression were also altered by CGP administration in untrained animals.

##### MEF2D protein and mRNA levels

**Fig. 5:**
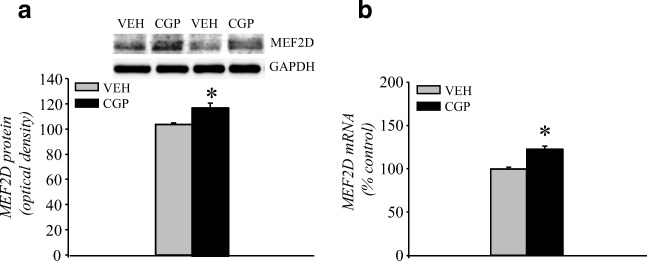
Myocyte enhancer factor 2D (MEF2D) protein and mRNA levels in the adult mPFC of postnatal CGP-treated untrained rats. MEF2D protein (**a**) and mRNA (**b**) levels. The MEF2D protein level was measured in the nuclear fraction of the mPFC. Photomicrographs show examples of the immunoblots of MEF2D and GADPH antibodies. Each data point represents the mean ± SEM; *n* = 6 per group. *p < 0.05 vs VEH (one-way ANOVA)

MEF2D immunoreactivity was measured in the nuclear fraction of the mPFC, and CGP administration increased the levels of MEF2D protein (*F*(1, 10) = 13.07, *p* < 0.005; Fig. 5a).

CGP treatment also increased the mRNA levels of MEF2D (*F*(1, 10) = 34.97, *p* < 0.0002; Fig. 5b).

##### Arc mRNA

**Fig. 6:**
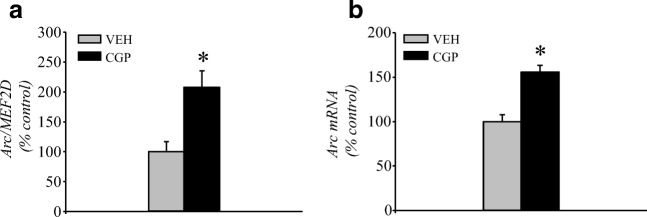
Activity-regulated cytoskeleton-associated protein (Arc) mRNA in the adult mPFC of postnatal CGP-treated untrained rats. Binding of MEF2D proteins to Arc DNA (**a**) and expression of Arc mRNA (**b**). Each data point represents the mean ± SEM; *n* = 5 (**a**) or *n* = 6 (**b**) per group. *p < 0.05 vs VEH (one-way ANOVA)

CGP significantly increased the amount of Arc DNA immunoprecipitated by MEF2D (*F*(1, 8) = 7.16, *p* < 0.03; Fig. 6a), and an increase in the mRNA level of Arc induced by CGP was also found (*F*(1, 10) = 31.26, *p* < 0.0003; Fig. 6b).

### Effect of early-life CGP treatment on epigenetic factors in the mPFC of adult (P65–P70) trained animals after memory retrieval in TFC

Epigenetic modifications are known to be involved in memory formation (Fischer et al. 2007, Jarome and Lubin 2014). Therefore, to determine, whether the deficit in fear memory in TFC paradigm previously observed in the CGP model (Latusz et al. 2017) might be related to changes in epigenetic factors induced by early-life blockade of NMDA receptor during memory retrieval in adulthood, the phosphorylation and acetylation of histone H3 and HDACs levels were investigated directly after memory test.

#### Histone modifications

##### Phosphorylation of histone H3

**Fig. 7:**
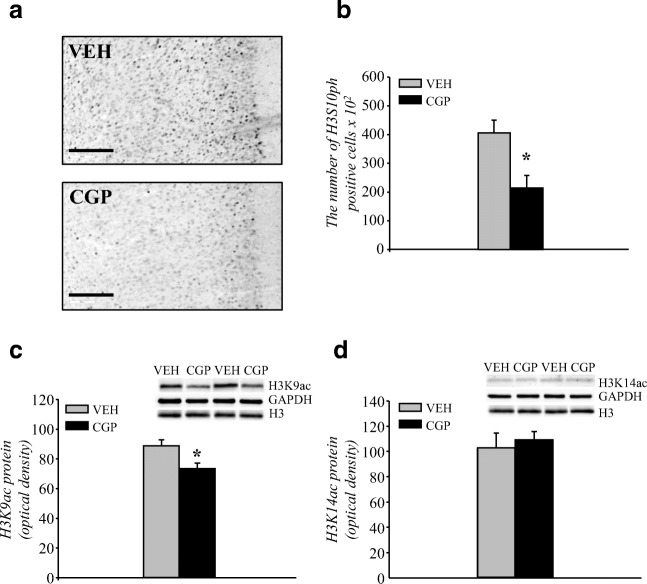
Histone H3 phosphorylation and acetylation in the adult mPFC of postnatal CGP-treated trained rats during memory retrieval in trace fear conditioning. Memory test was performed 24 h after training. The number of histone H3 phosphorylation at serine 10 (H3S10ph; **a**, **b**) and histone H3 acetylation at lysine 9 (H3K9ac; **c**) or at lysine 14 (H3K14ac; **d**) protein levels in the nuclear fraction. Photomicrographs show examples of the prelimbic part of the mPFC immunoprobed for H3S10ph (**a**) or the immunoblots of H3K9ac, H3K14ac, total histone H3 and GADPH antibodies (**c**, **d**). Each data point represents the mean ± SEM; *n* = 7 (**b**) or *n* = 6 (**c**, **d**). *p < 0.05 vs VEH (one-way ANOVA)

Histone H3 phosphorylation was studied using H3S10ph immunostaining and stereological analysis in the adult mPFC of trained rats (Fig. 7a). The number of H3S10ph positive cells was also affected in CGP-treated trained animals (treatment: *F*(1, 12) = 11.09, *p* < 0.006) during memory retrieval (Fig.7b), and it increased in VEH-treated trained rats almost 10 times compared to that in VEH-treated untrained rats (Fig. 1b). The number of H3S10ph nuclei in trained CGP animals appears to be at a similar level as that in CGP-treated untrained rats (Fig. 1b).

##### Acetylation of histone H3

Two forms of histone acetylation, H3K9ac and H3K14ac, were also studied in the nuclear fractions of mPFC of trained rats. CGP administration induced the changes in H3K9ac (treatment: *F*(1, 10) = 9.7, *p* < 0.01; Fig. 7c) but not in H3K14ac (treatment: *F*(1, 10) = 118.7, *p* = 0.613; Fig. 7d) protein level in the adult mPFC of trained animals.

#### Histone deacetylase

HDAC2 and HDAC5 were investigated during memory retrieval because of their involvement in memory formation (Agis-Balboa et al. 2013, Guan et al. 2009).

**Fig. 8:**
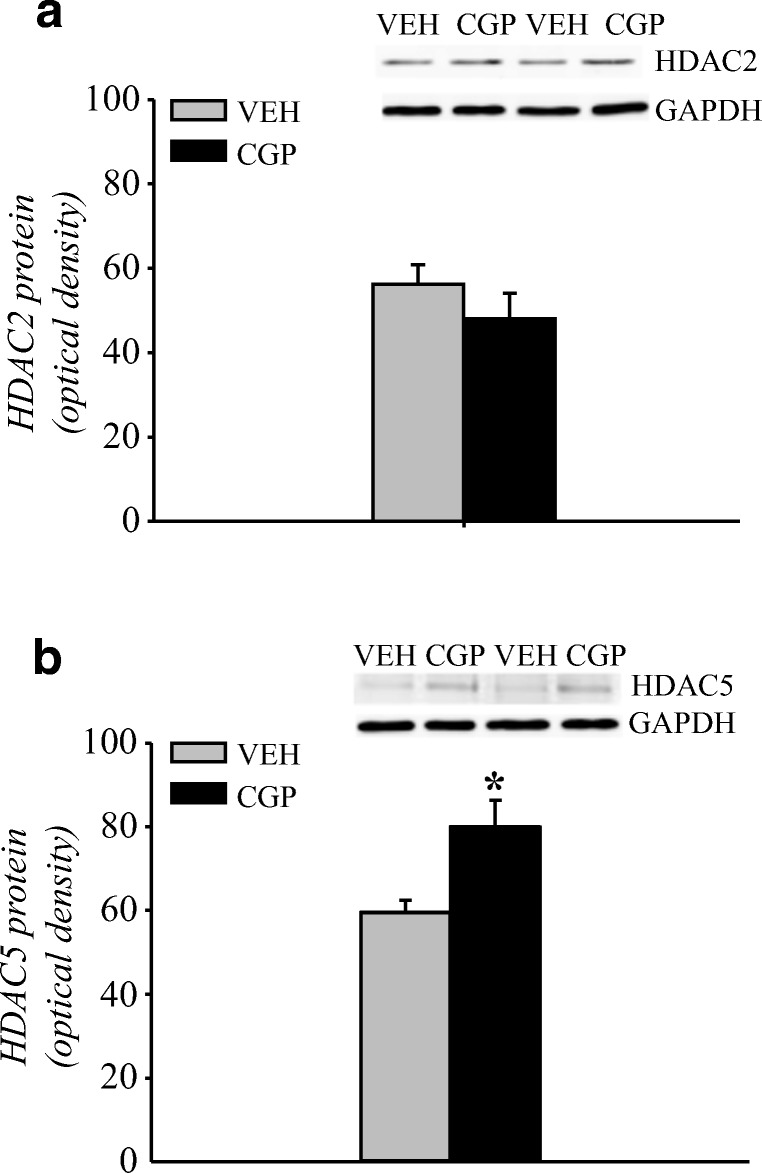
Histone deacetylase (HDAC) proteins in the nuclear fraction of the adult mPFC of postnatal CGP-treated trained rats during memory retrieval in trace fear conditioning. Memory test was performed 24 h after training. HDAC2 (**a**) and HDAC5 (**b**) protein levels. Photomicrographs show examples of the immunoblots of HDAC2, HDAC5 and GADPH antibodies. Each data point represents the mean ± SEM; *n* = 6. *p < 0.05 vs VEH (one-way ANOVA)

Both HDAC2 and HDAC5 protein levels were analysed in the nuclear fraction of the mPFC of trained animals. CGP administration did not alter HDAC2 (treatment: *F*(1, 10) = 1.4, *p* = 0.264; Fig. 8a) but increased HDAC5 (treatment: *F*(1, 10) = 10.42, *p* < 0.01; Fig. 8b) protein levels in the adult mPFC of trained animals.

### Effect of SB administration on the freezing response and epigenetic factors in the mPFC of adult (P65–P70) trained animals after memory retrieval in TFC

Our study (Figs. 7 and 8) showed that early-life CGP administration affected H3K9ac, H3S10ph and HDAC5 protein levels in the adult mPFC in trained animals after memory testing in TFC, but it did not change H3K14ac and HDAC2 levels under the same experimental conditions. Because of the decrease in H3K9ac and the increase in HDAC5 protein levels in the nuclear fraction of the adult mPFC of trained animals (Figs. 7 and 8), we performed experiments to investigate whether the non-selective HDAC inhibitor SB might prevent a deficit in the freezing response in the memory test in TFC. Based on the literature data showing an increase in the levels of neuronal activity markers (pERK protein, c-fos mRNA) in the mPFC between 0 and 4 h after training in the TFC (Han et al. 2003, Runyan et al. 2004), we decided to inject SB immediately (0 h) or 2 h after training.

### Effect of SB on the freezing response

There were no differences in the freezing response during the memory retrieval test between the VEH and CGP groups injected with NaCl either directly or 2 h after training; therefore, we combined the results from these animals into one VEH + NaCl or CGP + NaCl group, and the effects of SB administration either directly (SB 0 h) or 2 h (SB 2 h) after training were compared to these groups.

**Fig. 9:**
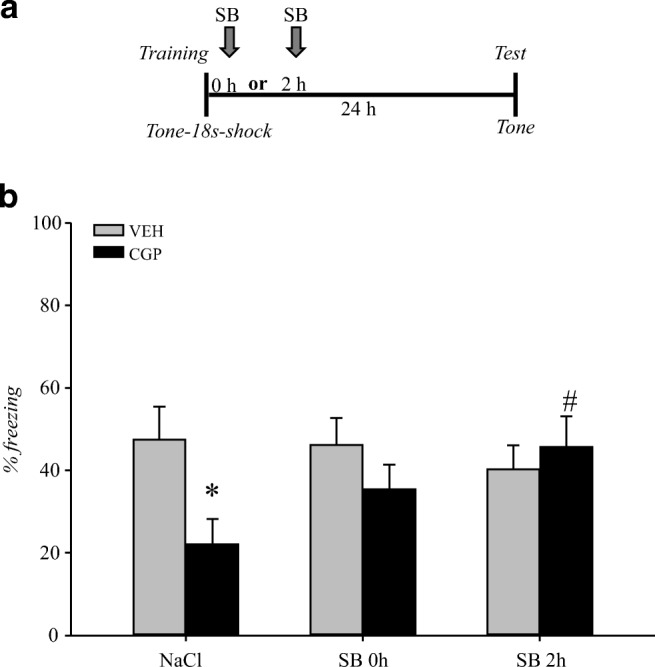
Effect of histone deacetylase inhibitor sodium butyrate (SB) on the decrease in freezing response induced by CGP postnatal treatment during memory retrieval in trace fear conditioning (TFC). Vehicle (NaCl) or SB was given immediately or 2 h after training (SB 0 h or SB 2 h, respectively), and a memory test was performed 24 h after training (**a**). The freezing response in TFC (**b**). Each data point represents the mean ± SEM; *n* = 12. *p < 0.05 vs VEH + NaCl group, ^#^*p* < 0.05 vs CGP + NaCl group (three-way ANOVA followed by Newman-Keuls test)

Early-life pretreatment with CGP induced changes in the freezing response (pretreatment: *F*(1, 88) = 9.7, *p* < 0.002), while treatment with SB in adulthood did not affect freezing (treatment: *F*(1, 88) = 2.5, *p* = 0.12). However, there was an interaction between CGP and SB administration (pretreatment × treatment: *F*(1, 88) = 6.38, *p* < 0.01), but that effect was not dependent on the time of SB injection after training (pretreatment × treatment × time: *F*(1, 88) = 0.8, *p* = 0.37). A significant decrease in the freezing level was observed only for the CGP + NaCl group (*p* < 0.05 vs VEH + NaCl group), and SB treatment reversed that decrease only when administered 2 h (*p* < 0.05 vs CGP + NaCl group) but not directly after training (*p* = 0.14 vs CGP + NaCl group, Fig. 9). SB administration did not influence the freezing response in any VEH group (*p* = 0.98 vs VEH + NaCl group).

At the next stage of our experiments, we performed a biochemical analysis in the mPFC. Because SB had a stronger effect on freezing response when administered 2 h after training instead of being administered immediately after training, in the biochemical experiments, 0.9% NaCl or SB was administered 2 h after training, a memory retrieval test was performed 24 h after training and the animals were sacrificed immediately after the test.

### Effect of SB on epigenetic markers

#### Histone modifications

##### Phosphorylation of histone H3 in memory test

**Fig. 10:**
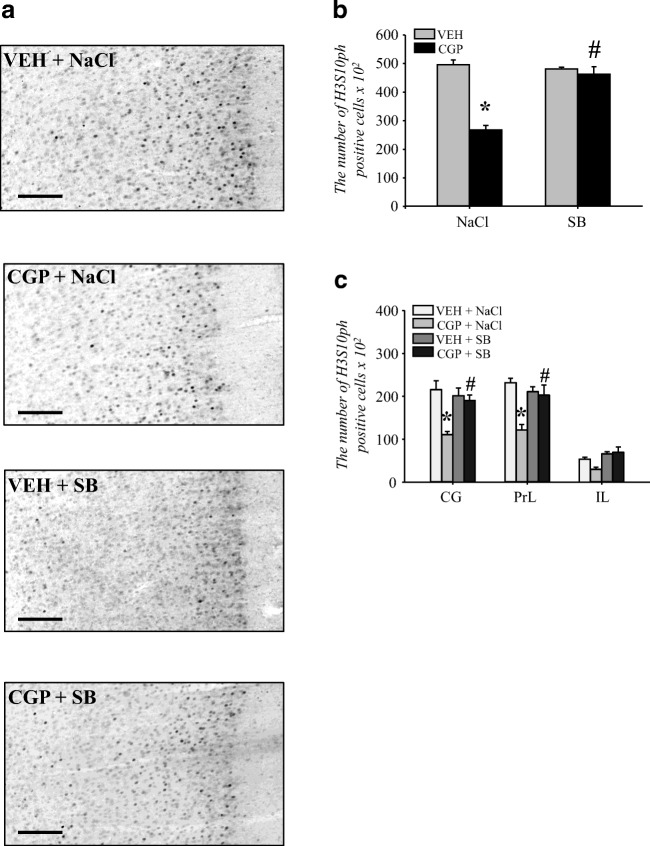
Effect of the histone deacetylase inhibitor sodium butyrate (SB) on histone H3 phosphorylation at serine 10 (H3S10ph) in the adult mPFC of trained rats during memory retrieval in trace fear conditioning. Vehicle (NaCl) or SB was given 2 h after training, and a memory test was performed 24 h after training. Light photomicrographs of the prelimbic part of the mPFC immunoprobed for H3S10ph (**a**) and examined for the number of immunopositive cells (**b**, **c**) are shown. The number of H3S10ph nuclei in the whole mPFC (**b**) and in the regions (cingulate (CG), prelimbic (PrL) and infralimbic (IL) cortices (c)). The scale bars represent 100 μm. Each data point represents the mean ± SEM; *n* = 5 per group. *p < 0.05 vs VEH + NaCl, ^#^*p* < 0.05 vs CGP + NaCl (two-way ANOVA (**b**) or two-way repeated measures ANOVA (**c**) followed by Newman-Keuls)

Changes in histone H3 phosphorylation induced by early-life blockade of the NMDA receptor were observed in the adult mPFC in trained animals (Fig. 7a, b). Therefore, the effect of SB on histone H3 phosphorylation and distribution in the adult mPFC of trained rats was studied using H3S10ph immunostaining and stereological analysis (Fig. 10a). During memory retrieval, the number of H3S10ph nuclei increased almost 10 times in the trained VEH + NaCl group (Fig. 10b) compared to that in untrained VEH-treated rats (Fig. 1b), and it was similar to the effect observed on Fig. 7b. Early-life pretreatment with CGP affected the number of H3S10ph nuclei (pretreatment *F*(1, 16) = 62.2, *p* < 0.000002), and adult treatment with SB also affected histone H3 phosphorylation (treatment = *F*(1, 16) = 30.52, *p* < 0.00005). There was also an interaction between these two factors (pretreatment × treatment = *F*(1, 16) = 47.99, *p* < 0.000003). CGP administration decreased the number of H3S10ph nuclei (*p* < 0.0002 vs VEH + NaCl; Fig. 10b), which appears to be at a similar level as that in untrained CGP-treated rats (Fig. 1b). However, SB administration inhibited the CGP-induced reduction in H3S10ph in trained animals (*p* < 0.0002 vs CGP + NaCl group), but SB treatment did not affect the VEH-treated group (*p* = 0.34 vs VEH + NaCl; Fig. 10b).

The number of H3S10ph cells in the subregions of the mPFC of trained animals was dependent on early-life manipulation (subregions × pretreatment = *F*(2, 32) = 4.62, *p* < 0.02) but not on SB administration (subregions × treatment = *F*(2, 32) = 0.068, *p* = 0.93). There was also no interaction between factors (subregions × pretreatment × treatment = *F*(2, 32) = 2.53, *p* = 0.096). A significant decrease in the number of H3S10ph cells was observed in cingulate (CG, *p* < 0.0002) and prelimbic (PrL, *p* < 0.0002) but not infralimbic (IL, *p* = 0.17) cortices in the CGP + NaCl group compared to that in the VEH + NaCl group (Fig. 10c). SB administration prevented the reduction in H3S10ph in both cingulate (*p* < 0.003 vs CGP + NaCl) and prelimbic (*p* < 0.002 vs CGP + NaCl) cortices. SB did not affect the number of H3S10ph cells in the VEH-treated group in any mPFC subdivision (*p* = 0.84 for CG, *p* = 0.39 for PrL, *p* = 0.46 for IL vs VEH + NaCl group; Fig. 10c).

##### Acetylation of histone H3

**Fig. 11:**
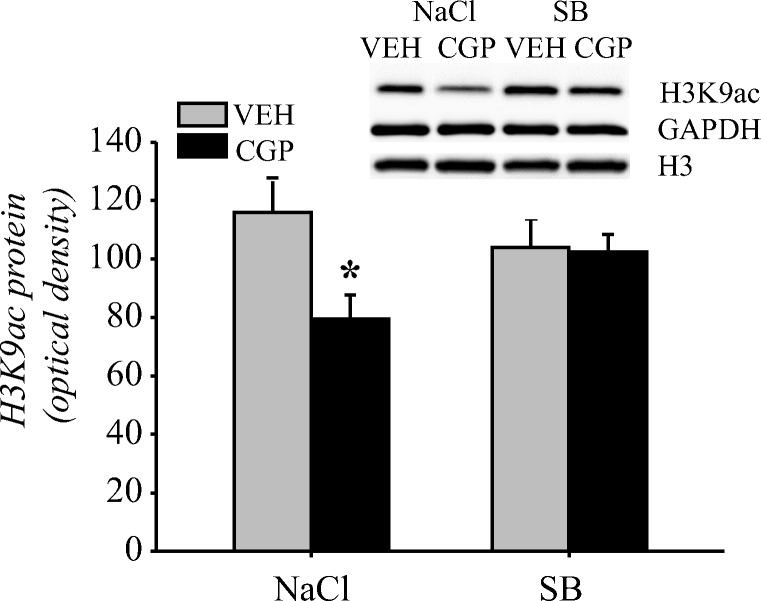
Effect of the histone deacetylase inhibitor sodium butyrate (SB) on histone H3 acetylation at lysine 9 (H3K9ac) in the nuclear fraction of the adult mPFC of trained rats during memory retrieval in trace fear conditioning. Vehicle (NaCl) or SB was given 2 h after training, and a memory test was performed 24 h after training. Photomicrographs show examples of the immunoblots of H3K9ac, total histone H3 and GADPH antibodies. Each data point represents the mean ± SEM; *n* = 6 per group. *p < 0.05 vs VEH + NaCl (two-way ANOVA followed by Newman-Keuls)

The effect of SB treatment on acetylation at H3K9 in the fear memory test was analysed due to our results showing the decrease in H3K9ac level in the adult mPFC of CGP-treated animals after memory retrieval in TFC (Fig. 7c). Early-life CGP treatment affected H3K9ac levels (pretreatment *F*(1, 20) = 5.82, *p* < 0.03), but SB administration did not have any effect (treatment = *F*(1, 20) = 0.46, *p* = 0.5). However, there was an interaction between factors (pretreatment × treatment = *F*(1, 20) = 4.96, *p* < 0.04). A statistically significant decrease in H3K9ac levels was found in the CGP + NaCl group (*p* < 0.02 vs the VEH + NaCl group; Fig. 11). SB administration prevented the decrease in H3K9ac in the CGP group; however, the effect did not reach statistical significance (*p* = 0.053 vs CGP + NaCl group, *p* = 0.45 vs VEH + NaCl group) and did not affect the VEH group (*p* = 0.29 vs VEH + NaCl group; Fig. 11).

##### HDAC5 and MEF2D protein levels

Our study showed an increase in HDAC5 levels in the nuclear fraction of adult mPFC of CGP-treated trained animals (Fig. 8b). Moreover, results demonstrated that early-life blockade of the NMDA receptor affected HDAC5 and MEF2D expression in the adult mPFC of untrained animals (Figs. 4 and 5). Therefore, the effect of SB administration on HDAC5, pHDAC5, and MEF2D levels was analysed in the adult mPFC of trained rats.

##### HDAC5 protein levels

**Fig. 12:**
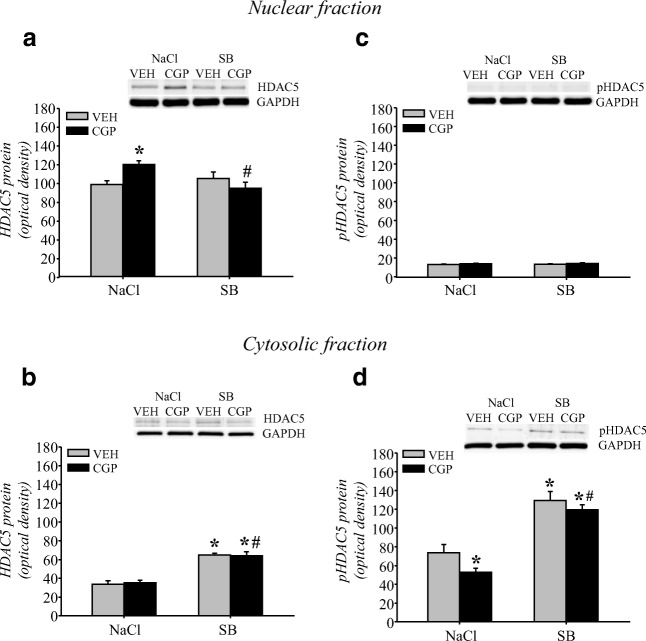
Effect of the histone deacetylase inhibitor sodium butyrate (SB) on histone deacetylase 5 (HDAC5) protein levels in the adult mPFC of trained rats during memory retrieval in trace fear conditioning. Vehicle (NaCl) or SB was given 2 h after training, and a memory test was performed 24 h after training. HDAC5 protein levels in nuclear (a) and cytosolic (b) fractions. Phosphorylated form of HDAC5 (pHDAC5) protein levels in nuclear (c) and cytosolic (d) fractions. Photomicrographs show examples of the immunoblots of HDAC5, pHDAC5 and GADPH antibodies. Each data point represents the mean ± SEM; *n* = 6 per group. *p < 0.05 vs VEH + NaCl, ^#^p < 0.05 vs CGP + NaCl (two-way ANOVA followed by Newman-Keuls test)

The levels of HDAC5 and pHDAC5 proteins in memory retrieval were analysed in nuclear and cytosolic fractions of the mPFC (Fig. 12a, b, c, d).

Neither early-life treatment with CGP nor adult manipulation with SB influenced HDAC5 protein levels in the nuclear fraction in the memory test (pretreatment = *F*(1, 20) = 1.16, *p* = 0.29) or (treatment = *F*(1, 20) = 3.54, *p* = 0.07). However, there was an interaction between these two factors (pretreatment × treatment = (1, 20) = 10.15, *p* < 0.005). An increase in HDAC5 levels during memory retrieval was observed in the CGP + NaCl group (*p* < 0.02 vs VEH + NaCl group), and SB administration abolished that effect (*p* < 0.008 vs CGP + NaCl group; Fig. 12a). SB did not alter the HDAC5 levels in the VEH group (*p* = 0.37; Fig. 12a). In the cytosolic fraction, postnatal treatment did not affect HDAC5 protein levels (pretreatment = *F*(1, 20) = 0.028, *p* = 0.87), but SB administration altered HDAC5 levels during memory retrieval (treatment = *F*(1, 20) = 118.8, *p* < 0.000001). However, there was no interaction between the analysed factors (pretreatment × treatment = *F*(1, 20) = 0.16, *p* = 0.69). SB administration significantly increased HDAC5 levels in both the VEH- and CGP-treated groups (*p* < 0.0002 vs VEH + NaCl and CGP + NaCl groups; Fig. 12b).

The level of pHDAC5 was very low in the nuclear fraction and was not changed in the memory test (pretreatment = *F*(1, 20) = 3.16, *p* = 0.09), (treatment = *F*(1, 20) = 0.16, *p* = 0.69) and (pretreatment × treatment = *F*(1, 20) = 0.009, *p* = 0.93; Fig. 12c). In contrast, early-life blockade of the NMDA receptor and adult treatment separately affected the pHDAC5 levels in the cytosolic fraction ([pretreatment = *F*(1, 20) = 5.76, *p* < 0.03] or [treatment = *F*(1, 20) = 90.38, *p* < 0.000001]). However, there was no interaction between these factors (pretreatment × treatment = *F*(1, 20) = 0.69, *p* = 0.42). A significant decrease in pHDAC5 was observed in the CGP + NaCl group (*p* < 0.04 vs VEH + NaCl group). SB administration increased the pHDAC5 levels in both the VEH- and CGP-treated groups (*p* < 0.0003 vs VEH + NaCl group, and *p* < 0.0002 vs CGP + NaCl group; Fig. 12d).

##### MEF2D protein levels

**Fig. 13:**
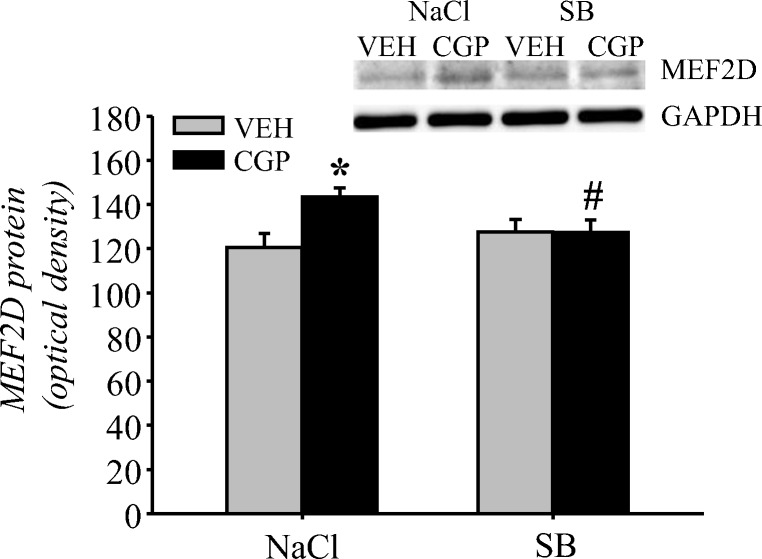
Effect of the histone deacetylase inhibitor sodium butyrate (SB) on myocyte enhancer factor 2D (MEF2D) protein levels in the nuclear fraction of adult mPFC of trained rats during memory retrieval in trace fear conditioning. Vehicle (NaCl) or SB was given 2 h after training, and a memory test was performed 24 h after training. Photomicrographs show examples of the immunoblots of MEF2D and GADPH antibodies. Each data point represents the mean ± SEM; *n* = 6 per group. *p < 0.05 vs VEH + NaCl, ^#^*p* < 0.05 vs CGP + NaCl (two-way ANOVA followed by Newman-Keuls test)

The MEF2D protein level was analysed in the nuclear fraction. Postnatal blockade of the NMDA receptor affected the MEF2D level in memory retrieval (pretreatment = *F*(1, 20) = 5.32, *p* < 0.04), but treatment during memory consolidation did not (treatment = *F*(1, 20) = 0.89, *p* = 0.36). However, there was an interaction between these factors (pretreatment × treatment = *F*(1, 20) = 5.7, *p* < 0.03). An increase in the MEF2D protein level during memory retrieval was observed in the CGP + NaCl group (*p* < 0.04 vs VEH + NaCl group), and SB administration prevented this effect (*p* < 0.05 vs CGP + NaCl group). However, SB did not influence MEF2D levels in the VEH group (*p* = 0.57 vs VEH + NaCl group; Fig. 13).

##### Arc mRNA

**Fig. 14:**
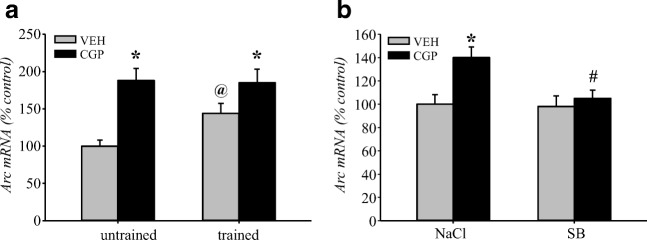
Activity-regulated cytoskeleton-associated protein (Arc) mRNA in the adult mPFC of postnatal CGP-treated rats. Arc mRNA levels were analysed in untrained (only home cages) and trained (after memory retrieval in trace fear conditioning) rats (**a**). The effect of sodium butyrate (SB) administration on Arc mRNA levels in trained animals (**b**). Vehicle (NaCl) or SB was given 2 h after training, and a memory test was performed 24 h after training. Each data point represents the mean ± SEM; *n* = 6 per group (**a**) and *n* = 5 per group (**b**). *p < 0.05 vs appropriate VEH group, ^@^*p* < 0.05 vs VEH-untrained group, ^#^*p* < 0.05 vs CGP + NaCl trained group (two-way ANOVA followed by Newman-Keuls test)

We observed that early-life CGP treatment increased Arc mRNA levels in the adult mPFC of untrained animals (Fig. 6b). We decided to compare the level of Arc mRNA between animals treated early with VEH or CGP without any experiments in adulthood (untrained animals) and similarly treated animals subjected to memory retrieval in TFC in adulthood (trained animals, Fig. 14a) and to determine whether memory retrieval might affect mRNA levels in the adult mPFC. Statistical analysis revealed that CGP treatment influenced the mRNA level (treatment = *F*(1, 20) = 22.43, *p* < 0.0002), but the memory factor did not (test = *F*(1, 20) = 2.29, *p* = 0.15). There was also no interaction between factors (treatment × test = *F*(1, 20) = 2.91, *p* = 0.10). Similar to previous results (Fig. 6b), there was a significant increase in Arc mRNA in untrained CGP-treated groups compared to that in the untrained VEH group (*p* < 0.02). During memory retrieval, the Arc mRNA levels increased in the trained VEH group compared to those in untrained VEH-treated rats (*p* < 0.04; Fig. 14a). However, the memory test did not affect the level of Arc mRNA in the CGP group, which remained at the same level as that in untrained CGP rats (*p* = 0.89), and was even higher than that observed in trained VEH rats (*p* < 0.05; Fig. 14a).

Because of the changes in Arc mRNA levels in trained rats, we also determined whether SB treatment might influence Arc mRNA levels during the memory test**.** The Arc mRNA level depended on either early-life CGP administration (pretreatment = *F*(1, 16) = 10.06, *p* < 0.006) or SB administration (treatment = *F*(1, 16) = 6.18, *p* < 0.03). There was also a statistical interaction between the analysed factors (pretreatment × treatment = *F*(1, 16) = 4.67, *p* < 0.05, Fig. 14b). Similar to the previously observed effect in trained animals (Fig. 14a), Arc mRNA levels were higher in the trained CGP + NaCl group (*p* < 0.005) than in the trained VEH + NaCl group. SB administration 2 h after training abolished the effect of CGP on Arc mRNA levels during memory retrieval (*p* < 0.0005 vs CGP + NaCl group, *p* = 0.64 vs VEH + NaCl group). However, SB treatment did not alter Arc mRNA expression in the trained VEH group (*p* = 0.82 vs VEH + NaCl group; Fig. 14b).

## Discussion

The present study indicates that the postnatal blockade of NMDA receptors during a critical developmental brain window (the first 3 weeks of postnatal life) (Snyder and Gao 2013) affected histone H3 phosphorylation but not acetylation in the adult mPFC of untrained animals. Early-life CGP administration was also found to alter HDAC5, MEF2D and Arc expression. Moreover, some epigenetic factors were changed during memory retrieval in TFC test and CGP treatment increased H3S10ph and HDAC5 levels, but it decreased H3K9ac in the adult mPFC of trained animals. Behavioural experiments using the TFC paradigm also showed that the deficit in fear memory retrieval observed in CGP-treated animals might be related to alterations in some epigenetic modifications in the adult mPFC. The administration of SB, an HDAC inhibitor (New et al. 2012), during memory consolidation not only blocked fear memory disruption induced by early-life NMDA receptor malfunction but also abolished epigenetic dysregulation in the adult mPFC of trained animals.

Early-life CGP administration enhanced histone H3 phosphorylation in the adult mPFC of untrained animals. A similar increase in the number of H3S10ph cells in the mPFC is also induced by a single injection of the NMDA receptor antagonist MK-801 in adulthood (Mackowiak et al. 2013), which is considered a pharmacological model of schizophrenia (Wedzony et al. 2000). However, there are no changes in histone H3 phosphorylation in the adult mPFC of rats treated with phencyclidine in adolescence (Aoyama et al. 2014). The above findings suggest that alterations in the phosphorylation level of H3 in the adult mPFC induced by dysfunction of the NMDA receptor are dependent on age. Increased phosphorylation of histone H3 occurs in response to activity-dependent signalling (Riccio 2010) and might suggest that early-life blockade of NMDA receptor induces continuous activity-dependent remodelling of chromatin to the transcription active state in the adult mPFC.

CGP administration did not affect the acetylation level of either H3K9 or H3K14 in the adult mPFC of untrained animals. However, changes in histone H3 acetylation were detected in the prefrontal cortex of schizophrenic subjects (Tang et al. 2011). Moreover, a decrease in H3K9ac but not H3K14ac was observed in the adult mPFC of animals with adolescent blockade of the NMDA receptor (Aoyama et al. 2014, Koseki et al. 2012). Thus, impairment of histone H3 acetylation in adulthood might also depend on the specific developmental stage at which dysfunction of the NMDA receptor occurs.

The results from clinical and preclinical studies of schizophrenia suggest that HDAC2 and HDAC5 are altered in prefrontal cortex (Aoyama et al. 2014, Gilbert et al. 2019, Koseki et al. 2012, Schroeder et al. 2017). HDAC2 and HDAC5 belong to two different classes of HDACs (HDAC I and HDAC IIa, respectively), and they are different in deacetylase activity and cellular distribution. HDAC2 has a higher deacetylase activity than HDAC5, and HDAC2 is localised mainly in the nuclear compartment in contrast to HDAC5, which can be found in both nuclear and cytoplasmic compartments (New et al. 2012). Our study did not show the effect of early-life CGP administration on HDAC2 protein levels in the mPFC of adult untrained rats, although changes in HDAC2 expression were observed in patients with schizophrenia (Gilbert et al. 2019, Schroeder et al. 2017). Thus, early-life dysfunction of the NMDA receptor might not affect HDAC2 in adulthood. However, alterations in HDAC5 expression were observed in the adult mPFC of untrained animals. HDAC5 is an epigenetic modifier that shows minimal deacetylase activity, although it has a highly conserved catalytic domain; moreover, it has been suggested that HDAC5 acts as an adaptor of repressor complexes, rather than as an enzyme (Parra 2015). The transcriptional function of HDAC5 is regulated by its subcellular localization, which is specified by neuronal activity (Chawla et al. 2003). NMDA receptors are involved in the regulation of the nucleocytoplasmic shuttling of HDAC5, and the activation of synaptic NMDA receptors induces HDAC5 translocation to the cytoplasm, whereas the stimulation of synaptic and extrasynaptic NMDA receptors inhibits nuclear export (Chawla et al. 2003). Thus, HDAC5 nuclear export provides a mechanism for input-specific gene expression, since HDAC5 is a transcriptional co-repressor that binds to MEF2 proteins in the nucleus and thereby represses MEF2-mediated transcription (McKinsey et al. 2000, McKinsey et al. 2001). HDAC5 translocation to the cytoplasm is connected with its phosphorylation (McKinsey et al. 2000, Parra and Verdin 2010). Our results showed that early-life changes in NMDA receptor function affected the regulation of HDAC protein levels in the nucleus, and a decrease in HDAC5 protein levels in the nucleus without any changes in the cytoplasm was observed in the adult mPFC of the CGP group. Moreover, a decrease in the phosphorylated form of HDAC5 after CGP treatment was also found in the cytoplasm. These results indicate that the decrease in HDAC5 protein levels in the nucleus was probably not related to the phosphorylation level and translocation to the cytoplasm but rather to the decrease in total HDAC5 protein levels mainly observed in the nucleus. The lack of changes in HDAC5 in the cytoplasmic compartment of CGP rats might suggest that dephosphorylation of pHDAC5 might be increased and the cytoplasmic accumulation of HDAC5 might be considered a negative regulator of HDAC5 function in the nucleus (Di Giorgio and Brancolini 2016). The early postnatal blockade of NMDA receptors induced an increase in the HDAC5 mRNA levels in contrast to the HDAC5 protein levels, which might also be considered part of a negative feedback loop that regulates HDAC function in the nucleus (Di Giorgio and Brancolini 2016). Thus, the early-life blockade of NMDA receptors induced a decrease in HDAC5 protein levels that was not compensated by the increase in HDAC5 mRNA levels. Changes in HDAC5 protein levels in the adult mPFC induced by adolescent blockade of NMDA receptors have also been reported by other researchers (Aoyama et al. 2014, Koseki et al. 2012). However, these findings showed an increase in HDAC5 protein levels in the nucleus and a decrease in cytosolic fractions of the adult mPFC (Aoyama et al. 2014, Koseki et al. 2012). It appears that impairment in HDAC5 regulation might be important for abnormalities in the mPFC induced by NMDA receptor dysfunction; however, the effect on HDAC5 level might depend on the age at administration of NMDA receptor antagonists, and the increase in nuclear HDAC5 levels (Aoyama et al. 2014, Koseki et al. 2012) might indicate the repression of gene transcription, while our results suggest an enhancement of transcriptional activity.

The last observation might be supported by an increased level of MEF2D proteins, since HDAC5 binds to MEF2 proteins in the nucleus (Parra 2015). The decreased HDAC5 protein levels in the nuclear fraction of the adult mPFC of the untrained CGP group were probably related to an increase in the MEF2D protein and mRNA levels. These findings suggest that the postnatal blockade of NMDA receptors might increase MEF2D transcriptional activity by enhancing MEF2D mRNA and protein levels. MEF2 targets genes that function in the nucleus and at the synapse (Flavell et al. 2008), HDAC5 is one of the MEF2 target genes that encode proteins that function within the nucleus to regulate transcription (Flavell et al. 2008), and MEF2 proteins bind to the HDAC5 proximal promoter. Thus, HDAC5 transcription may be under the control of MEF2 as a part of a negative feedback loop (Di Giorgio and Brancolini 2016). On the other hand, MEF2 directly regulates the expression of a large set of activity-regulated genes in neurons, i.e., Arc (Flavell et al. 2008). Our results showed an increase in Arc mRNA levels in the mPFC of the untrained CGP group, which was connected to an increase in MEF2D protein binding to the Arc gene. These results confirm findings that Arc expression is regulated by the MEF2D transcription factor (Flavell et al. 2008). An increase in Arc mRNA was also found in the mPFC after repeated phencyclidine treatments in adulthood (Thomsen et al. 2010). Thus, the blockade of NMDA receptors induces alterations in Arc mRNA levels in the mPFC; however, only our results showed that the early-life blockade of NMDA receptors might have long-lasting effects on Arc expression in the mPFC. Our results also suggest that CGP administration during the first 3 weeks of rat life increased transcriptional activity in the adult mPFC by affecting the level of epigenetic regulators. Thus, early-life dysfunction of the NMDA receptor might enhance prefrontal cortex activity, as was observed by others in human and animal studies. Human fMRI studies have shown that NMDA receptor antagonists increase metabolic activation in the prefrontal cortex (Breier, 1997, Vollenweider 1997). In animal models, NMDA receptor antagonists increase the spiking of spontaneously active neurons (Jackson et al., 2004) and activate c-fos expression in the neurons of the adult cortex (Kargieman, 2007).

In the present study, we used the TFC paradigm to evaluate the role of epigenetic abnormalities in the mPFC during fear memory retrieval in CGP animals. The present results are similar to those of our previous study (Latusz et al. 2017) and showed that early-life CGP administration induces fear memory deficits in adulthood in TFC. Several findings suggest that trace fear memory is dependent on prefrontal cortex function (Han et al. 2003, Runyan et al. 2004) and is regulated by epigenetic mechanisms (Sui et al. 2012). In detail, intra-mPFC infusion of SB enhanced memory formation in TFC; however, the effect was observed only in the long-term retention test (Sui et al. 2012). Our results showed that acute systemic SB administration during a specific window of memory consolidation (2 h but not immediately after training) prevented fear memory disruption induced by early-life blockade of the NMDA receptor. Although there are results showing that sustained SB treatment (4 weeks) facilitates learning and restores fear memory (Fischer et al. 2007), our behavioural studies demonstrated that a single SB injection given at a specific time during memory consolidation was sufficient to prevent the development of fear memory impairment induced by early-life blockade of the NMDA receptor, but it had no effect on control animals.

The effect of SB on the freezing response in memory retrieval of the trained CGP group might suggest that the acetylation level of histone might be involved in memory formation. The last observation is in line with literature data showing that histone acetylation is important for memory formation (Fischer et al. 2007, Jarome and Lubin 2014). Moreover, our study also showed a decrease in H3K9ac protein in adult mPFC after memory retrieval in the trained CGP group in contrast to the lack of changes in H3K9ac induced by CGP treatment in the adult mPFC of untrained animals. The discrepancy in the effect of CGP treatment on H3K9ac protein levels in the mPFC of untrained and trained animals might be related to changes in H3K9ac in the trained VEH group responding to memory formation in contrast to untrained home cage rats. We did not compare the H3K9ac level between untrained and trained VEH-treated animals; however, the alterations in the level of another histone modification activating gene transcription, i.e., H3S10ph, in the adult mPFC of VEH-treated groups suggest similar changes in H3K9ac levels. Thus, it appears that CGP prevents the learning-induced increase in H3K9ac. A decrease in H3K9ac levels in the CGP group in memory retrieval was abolished by SB administration. Thus, the behavioural effect of SB might be related to the H3K9ac level, which is in line with results showing that normalisation of H3K9ac in the mPFC might prevent schizophrenia-like abnormalities induced by adolescent blockade of the NMDA receptor (Aoyama et al. 2014, Koseki et al. 2012). However, SB administration did not affect H3K9ac in the trained VEH group, possibly because memory retrieval itself increased H3K9ac to the level that an effect of SB might not be observed.

We also analysed the involvement of other epigenetic regulatory mechanisms in the mPFC in memory formation. We noticed that memory retrieval induced a robust increase in the number of H3S10ph cells in the mPFC of postnatal VEH-treated rats. However, the number of phosphorylated histone H3 cells did not change in trained CGP rats, and the memory test remained at a similar level as in untrained CGP rats not subjected to a TFC procedure. The above findings suggested that memory retrieval induced a nucleosomal response and that this effect was not observed in the trained CGP group, probably because the active chromatin state was already observed in these animals. To the best of our knowledge, this study is the first to demonstrate that memory retrieval in trace fear memory increases histone H3 phosphorylation in the mPFC, although an increase in H3S10ph in the hippocampus in contextual fear conditioning has been reported by others (Besnard et al. 2014, Chwang et al. 2006). Moreover, the basal level of H3S10ph in the mPFC might be crucial for proper memory formation, and an excessively high level of histone H3 phosphorylation before memory encoding might be a factor disrupting memory formation. It also seems that the epigenetic events during memory consolidation are an important mechanism for memory storage, since SB administration during that period increased the number of H3S10ph cells in the CGP group in the memory test that corresponds to the behavioural response. The above effect of SB is probably a result of cross-talk between histone phosphorylation and increased acetylation (Sawicka and Seiser 2014). However, SB did not influence histone H3 phosphorylation in trained control animals. The last observation indicates that SB administration during memory consolidation normalises the impairment of histone phosphorylation induced by early-life blockade of NMDA receptors.

We also studied the level and cellular distribution of HDAC5 related to memory retrieval. We found an increase in HDAC5 protein in the nuclear compartment and a decrease in pHDAC5 in the cytoplasm in trained CGP-treated rats that might impair transcriptional activity. SB administration normalised HDAC5 levels in the nuclear fraction of CGP-treated rats; however, it increased both HDAC5 and pHDAC5 in the cytosolic fraction of both VEH- and CGP-treated animals. Our results suggest that SB administration during memory consolidation increases HDAC5 phosphorylation and export to the cytoplasm, which might be a mechanism regulating HDAC5 levels in the nucleus, particularly in the CGP group.

However, we also detected an increase in MEF2D protein in CGP rats during the memory test, which might be responsible for the memory deficit in these animals. Our results are in line with published data indicating that MEF2 overexpression impairs the formation of both spatial and fear memory (Cole et al. 2012). The important regulation of MEF2-mediated transcription is MEF2 phosphorylation (at serine 444 for MEF2D), and memory formation might be related to the phosphorylation level of MEF2 (Cole et al. 2012). We did not measure the phosphorylation level of MEF2D in our experiments; thus, we cannot rule out the possibility that deficits in memory retrieval in the CGP group in the TFC test might also be related to impairments in MEF2D phosphorylation. SB administration decreased MEF2D protein in the trained CGP group to the control level, which might be connected with a decrease in nuclear HDAC5 levels in these rats. Regulation of MEF2D levels by SB treatment might cause normal memory formation in CGP rats, since a decrease in MEF2 facilitated memory processes (Cole et al. 2012).

Our study showed Arc mRNA activation in the mPFC of VEH animals during memory retrieval; however, Arc mRNA activation was lower than that in the trained CGP group, which remained at the same level as in untrained CGP rats. MEF2D is known to regulate Arc expression, and a high level of Arc mRNA might be related to the higher level of MEF2D in this group. The SB effect on Arc mRNA seems to support this suggestion, since SB normalised both MEF2D protein and Arc expression in CGP-treated rats during memory retrieval. Some evidence also suggests that MEF2 overexpression might prevent memory formation by activating Arc transcription (Carmichael and Henley 2018) and indicates an important role of Arc in memory consolidation (Czerniawski et al. 2011, Plath et al. 2006). Moreover, the regulation of Arc expression appears to be important for the emergence of schizophrenia-like abnormalities (Manago and Papaleo 2017). Some genetic evidence indicates that Arc might be a risk gene for schizophrenia (Chuang et al. 2016, Huentelman et al. 2015), and a decrease in Arc mRNA was detected in the prefrontal cortex in individuals with schizophrenia (Guillozet-Bongaarts et al. 2014). The epidemiological and clinical data are supported by animal studies indicating that the genetic disruption of Arc induces a schizophrenia-like phenotype in mice (Manago et al. 2016). Our study showed an increase in Arc transcription in the mPFC of animals with schizophrenia-like abnormalities and provided some evidence that Arc overexpression might also be involved in some cognitive dysfunction.

SB as an HDAC inhibitor should reactivate epigenetically silenced genes by increasing histone acetylation; however, some in vitro and in vivo studies indicated that SB could also downregulated genes (Kratsman et al. 2016, Rada-Iglesias et al. 2007). It is suggested that SB-induced repression of gene expression might be related to a decrease of histone acetylation at gene promoters (Rada-Iglesias et al. 2007) or might be due to downstream effects by affecting transcription factors regulating gene transcription (Kratsman et al. 2016). In our study, we observed that SB decreased Arc mRNA in the CGP-trained group. Although the exact mechanism of SB action on Arc mRNA needs to be determined in further studies, it seems that SB effect is not related to an increase in H3K9ac level in CGP + SB-trained rats but most likely is related to a decrease in MEF2D protein level in this group. Thus, it appears that SB might affect fear memory by regulating transcription factors, rather than increasing histone acetylation.

Our results indicate that early-life dysfunction of NMDA receptors might change the epigenetic status in the adult mPFC and impair epigenetic control of the transcription activity of some genes, i.e., Arc. The above alterations might be involved in cognitive impairments related to memory formation in adulthood and support the observation that the regulation of epigenetic mechanisms by histone deacetylase inhibitors might be used to normalise memory processes (Campbell and Wood 2019).
